# Proposal for a Novel Abrasive Machining Method for Preparing the Surface of Periarticular Tissue during Orthopedic Surgery on Hip Joints

**DOI:** 10.3390/jfb12030050

**Published:** 2021-09-08

**Authors:** Paweł Zawadzki

**Affiliations:** Faculty of Mechanical Engineering, Poznan University of Technology, Maria Sklodowska-Curie Square, 60-965 Poznan, Poland; pawel.zawadzki@put.poznan.pl

**Keywords:** cartilage and bone machining, cutting forces, coefficient of friction, grinding mechanics

## Abstract

Drilling, cutting, and milling are the most common methods used in orthopedic surgery. However, popular machining methods do not obtain the complex shape of the periarticular tissue surfaces, increasing operation time and patient recovery. This paper reports an attempt to research a novel design of a machining process for surgical procedures. A device using abrasion machining based on mechanical erosion was proposed. Machining uses an undefined geometry of the cutting grains to cut tissue in any direction during oscillatory tool movement. This new concept is based on a cylindrical abrasive device made of brown fused alumina and silicon carbide grains deposited with an epoxy resin binder on the surface of a polyamide shaft. The best results in terms of machining efficiency were obtained for grains of the BFA80 type. Cutting experiments with different values in terms of cutting speed, granulation of the abrasive grains, pressure forces, and machining scope showed that the proposed concept, by developing the shape of the device, allows for penetration of the tissue structure. The research shows the possibility of using the proposed method during periarticular tissue machining.

## 1. Introduction

One of the basic procedures used during orthopedic procedures is the surface machining of cartilage and bone tissue [1]. This procedure is used in many orthopedic operations, although no detailed analysis of these processes in terms of machining properties has been carried out. Simple processing methods are commonly used. Lee et al. [2] carried out the research on bone tissue drilling and conducted experimental and simulation analyses of the thrust and torsion mechanical force model during bone drilling. The research focused on drill-bit parameters, cutting conditions, and cutting geometry while taking the material and friction properties into account through empirical specific energies. In a study by Augustin et al. [3], attention was paid to the thermo-necrosis effect accompanying all living tissue treatments. A similar problem was pursued by Amewoui et al. [4] by preparing a model that can predict bone temperature during drilling operations. Experimental validation showed that the model satisfactorily reproduces the temperature increase to the maximum value while overestimating the temperature of the cooling phase. Bai et al. [5] also discussed the temperature issue, who developed vibration-assisted drilling (VAD) methods for the temperature increase during bone drilling. This experiment indicated that the unfavorable thermal conditions of UVAD were caused by the higher applied frequency, which created a more significant amount of friction heat. Thermal necrosis can cause permanent damage to the circulatory and nervous system tissues, leading to their death. The surgical procedure may result in complications that lead to transplant rejection by the body or endoprosthesis [6]. A more complex issue concerning the control of the drilling process mechanic was presented by Wang et al. [7]. This research shows a method of detecting bone conditions during the drilling process based on a multi-sensor system. As the authors point out, the future of drilling will focus on intelligent processing based on an accurate recognition of the tissue’s structure and condition. Drilling is mainly used during joining bone fragments, fixing implants, or immobilizing the bone [8]. Another procedure is tissue cutting, about which James et al. [9] conducted extensive research presenting an analytical model for predicting bone sawing forces, which was then compared with experimental tests in which the bone was cut linearly at a speed of 2600–6200 mm/s, at a depth of 2.5–10 um, at which the resultant forces ranged from 8 to 11 N. Yaping et al. [10], when preparing the simulator for bone tissue cutting, conducted extensive experimental and simulation tests, showing the value of the forces accompanying cutting at the level of 4–7 N. Research on saw cutting directed, similar to drilling, toward the heat emission process was carried out by James et al. [11] using the sagittal saw. This study aimed to determine the effect of the applied thrust force and blade speed on generating heat. The 15 and 30 N forces were involved, with blade oscillation rates of 12,000 and 18,000 CPM. In histopathological examinations, the cut temperature reached 109 °C, which showed a wide increase in the necrotic zone of up to 0.75 mm from the cut site due to thermocrossing. Yan et al. [12], in preparing diamond wire sawing, indicated that wire saw cutting provides a shallow cutting depth that effectively achieves a malleable material removal mode. Research to counteract the thermal effects during the cutting process was conducted by Gwenllian et al. [13], comparing oscillating saws to burrs in temperature generation and histologic damage. The main focus was to compare the incision with and without water, and the result was that bone irrigation during resection could prevent bone necrosis. Bone cutting is mainly used to remove damaged tissue fragments, implantation of endoprostheses, and other orthopedic procedures. Another method is milling [1], which is discussed in more detail by Liao et al. [14]. The article presents innovative mechanistic models of cutting force and temperature for bone milling. It has been shown that the cutting force for the single cutting-edge tool depends on the thickness of the chip. A decrease in thermal necrosis penetration depth with an increase in cutting speed was also shown. Despite the growth in cutting speed, the temperature remains the same due to the rise in global heat flux. Similar results were shown by the work of Chen et al. [15] in which the resultant milling force was obtained at the level of 2–14 N. A detailed description of the milling process is presented by Qasemi et al. [16] in which the author uses a flat cutter to mill a beef bone by introducing specific machining parameters. The main research result was the determination of the surface roughness dependence on the cutting speed, direction, tool size, and feed. One study focused on assessing surface quality as an essential parameter of bone tissue treatment. Research focusing on evaluating the cutting force of bone tissue using FEM was presented by Liu et al. [17], with similar results to other authors. Depending on the cutting speed value (600–1000 mm/s), cutting force was at the level of 11–12 N. Depending on the cutting depth (0.1–0.5 mm), the increase in cutting force was more significant and amounted to 5–15 N. These results indicate that the FEM analysis reflects the actual experimental results. The last mechanical procedure used during the treatment of bone and cartilage tissue is surface abrasion. However, there is not much research on this issue. In most cases, they deal with thermal effects causing the necrosis phenomenon, as in the work of Mizutani et al. [18], where the diamond wheel was used with a coolant. Research has shown that increasing the amount of coolant during the abrasion process reduces the heat generated. A similar analysis using a spherical diamond burr was performed by Zhang et al. [19] by cutting the bone tissue’s surface, indicating that the amount of generated heat might cause bone osteonecrosis. Thermography was used in both studies focused on evaluating the influence of mist cooling and the direction of abrasion on the process temperature. It has been shown that, with a backward abrasion motion, the cryogenic saline mist could provide a better cooling performance than conventional flood irrigation. As can be seen, in the case of cutting by abrasion, scientists focus mainly on thermal effects, although it should be noted that the methods primarily used focus on craniofacial surgery.

The above methods result from certain preliminary assumptions regarding treating tissue methods, resulting from its damage or disease type. The most prevalent causes of treatment of periarticular tissues include cartilage and osteochondral lesions caused by trauma or other pathologies [20] and arthritis/osteoarthritis (or degenerative joint disease) [21]. Both cases most often concern articular surfaces or joint areas exposed to the most significant mechanical loads and are susceptible to damage due to their complexity. Currently, there are many methods of treating damaged articular surfaces: arthroscopic and open repair procedures [22], soft tissue grafts [23], osteochondral allograft transfer [24], autologous chondrocyte transplantation [25], autografts and allografts [21], and the most common total and partial joint replacements [21]. However, they require prior preparation of the articular and periarticular surfaces to perform the procedure [26,27]. Due to the popularity of standard surgical procedures, such as total femoral head resection [28] or total knee arthroplasty [29], there is no change in the processing technology or in the availability of specialized equipment for other processing methods. The processing procedures mentioned above, commonly used today, in many cases, require modernization by the appearance of new types of endoprostheses [30] or new methods used in tissue engineering. It should be noted that the interference in the joint structure is minimized by the use of surface endoprostheses [28,30] and the introduction of new solutions in the form of scaffold-based techniques [22]. If there is a minimization of the measures used, attention should also be paid to new treatment methods that will ensure the appropriate quality of the operation, not endanger the patient’s health, minimize interference, and speed up the procedure. Erosion machining based on abrasion is a technology that could prepare the precise geometry of the articular surfaces and other fragments of the skeletal system. Abrasion is a complex material removal operation involving cutting, rubbing, and ploughing between the grain and the machined material [31]. This process can be classified into Mold and Finish Abrasion (FFG) and Material Removal Abrasion (SRG). The SRG goal is to obtain a high removal rate, and FFG is performed to achieve the required form, finish, and accuracy [32]. The abrasion process is comparable to the milling process, but on a microscale. However, the phenomenon of friction on the clearance face of the grains should be taken into account. In abrasion, the ratio of tangential forces to normal forces is approximately 0.3–0.5, characteristic of the sliding friction process. This result shows that cutting grains is a small fraction concerning the abrasive grains [33]. The abrasion operation is characterized by a high surface finish and accuracy [34,35], the possibility of processing hard materials (bone tissue) [36,37,38] and obtaining highly accurate dimensions [39], less load pressure applied on machined material [40], and the possibility of machining a complex shape in the case of a smooth surface [41,42].

However, there are no new technologies related to articular surfaces machining, ensuring a consensus between all the requirements. Therefore, this study focused on developing a proprietary method of treating the hip joints’ articular and periarticular surfaces, focusing on abrasive treatment to remove diseased or damaged tissue. The solution included preparing an abrasive tool, which will allow one to obtain a specific shape following the operational assumptions under the influence of particular kinematics and processing parameters. The research included cutting forces, the coefficient of friction between the tool and the tissue, and the temperature accompanying the process. Furthermore, the tool wear mechanisms were indicated, its service life was determined, and two types of abrasive grains with different grain sizes were compared. The tissue structure’s influence on the machining process was also analyzed, and an analytical model was prepared to define the cutting parameters. The obtained results provide, for the first time in the literature, essential information regarding the erosion–abrasion performance of cartilage and bone tissue and selecting effective machining parameters.

## 2. Materials and Methods

### 2.1. Plan of the Experiment

**Figure 1:**
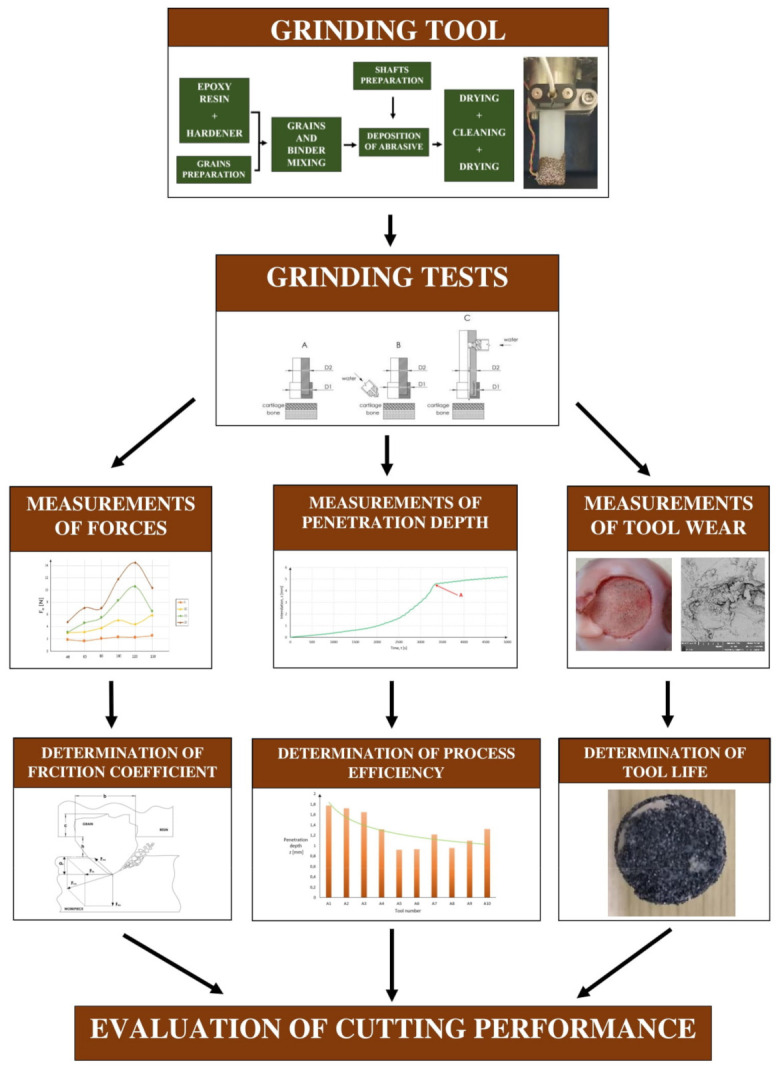
The scheme of a conducted experiment.

The primary cutting parameters of bone and tissue cartilage were evaluated as part of this study. Measurements of cutting forces and an evaluation of the friction coefficient were performed during the experiments. Therefore, the penetration depth and grinding efficiency were applied. The tests were supplemented by the performance of complete tribological tests, including grinding tool wear analysis. Figure 1 presents the overall scheme of the conducted experiments.

### 2.2. Manufacturing of Abrasing Tool

The silicon carbide (SiC) and brown fused alumina (BFA) abrasive tools were manufactured during the four-step process divided into manufacturing the product and preparing tools of a specific shape. In the first stage, a binder consisting of a styrene-modified epoxy resin with a hardener (triethyleneteraamine) in a ratio of 1:10, supporting the process of binding the substrates, was prepared. Additionally, the grains were prepared by checking for any impurities. In the next step, the grains and the binder were combined. Furthermore, polyamide (PA6) rollers with a diameter of 12 mm and a length of 50 mm were prepared. The rollers’ fronts and side surfaces (5 mm high) were covered with mixed resin and grains in the same step. They were subjected to intermediate drying, re-coated with a binder, and applied with abrasive tools. On each of the rollers, abrasive tools with a specific granulation were used. In this way, the abrasion tools prepared were subjected to shape correction and then dried at a temperature of 20 degrees for more than 200 min (indicated by the manufacturer). The abrasive tools were then transferred to an ultrasonic cleaner, cleaned in water, and then dried. During the research, six sizes of SiC (95–98% SiC) and BFA (94.5–97% Al_2_SO_4_) grains were used (see Table 1). It was assumed that the tools’ target movement would be in the range from 0.25 to 1 mm; therefore, the above grain sizes were considered significant. These grain sizes were adopted because, since the target range of the movement is from 0.25 to 1 mm, a single grain must move at least by the length of its diameter during this movement. Additionally, the size of the osteon varies between 0.25 and 0.35 mm in diameter. Therefore, it was recognized that the grain size must be close to the size of the osteon to perform precise machining. The indicated grain diameters should ensure the correct quality of the treated surface.

### 2.3. Machining Tests 

**Figure 2:**
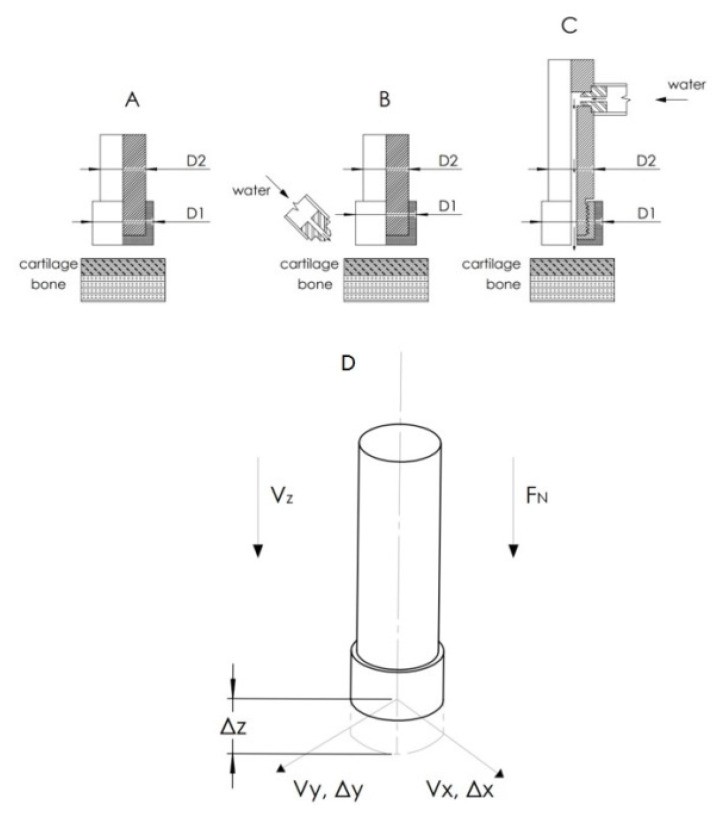
Methods of the abrasion process experiment: (**A**) dry abrasion, (**B**) wet abrasion with external irrigation, (**C**) wet abrasion with internal irrigation, and (**D**) kinematic of the abrasive tool movement and force load.

During studies, samples of pig femur heads, the femur lateral and medial condyle, and the tibia lateral and medial condyle taken from fresh bone material were used (Carrefour Polska Sp. z o.o., Poznan, Poland). All parts were supplied in natural anatomical forms, which reflected the natural processing conditions. Specimens were subjected to machining tests with specific motion kinematics in dry and wet cutting conditions, using a precise UMT Bruker Tribometer equipped with a 2-dimensional force sensor DFM-20 with a measurement range of 2–200 N and a resolution of 10 mN. During the tests, the abrasive tool made an oscillating motion f with a specified velocity in two directions (X and Y) with a constant load *F_N_* directed perpendicularly to the sample surface (see Figure 2D). The geometry of an abrasive tool made of SiC and BFA grains is presented in Figure 2A–C and Table 2. The parameters applied during the test are shown in Table 3. Selection of the cutting speed and load force values was based on orthogonal bone cutting recommendations [14,15]. 

During the tests, to simulate environmental conditions, water was injected between the abrasive tool and the tissue using a peristaltic pump system and a dedicated attachment (see Figure 2B,C) with external or internal irrigation. Washing with water was also intended to clean the surface of the cut tissue that could contaminate other tissue spaces during the operation.

### 2.4. Measurement of Abrasion Process Dynamics

**Figure 3:**
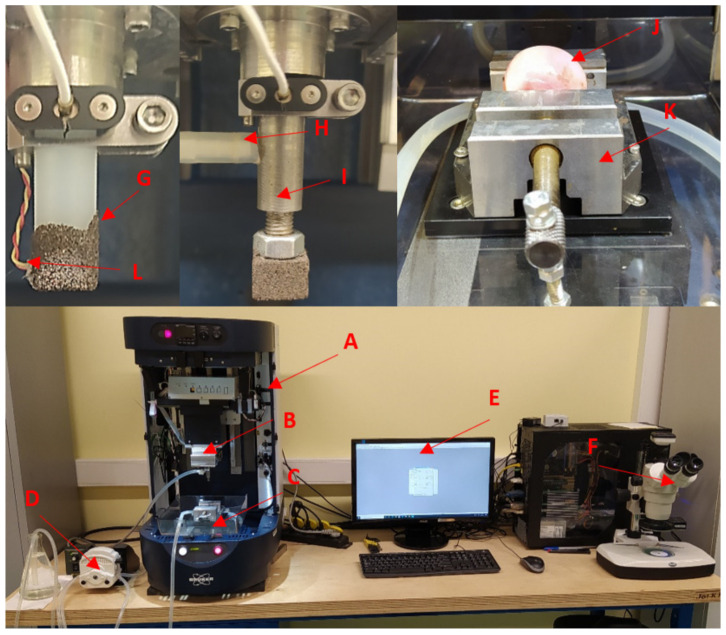
The experimental set-up for abrasion process dynamics evaluation: (A) Tribometer Bruker UMT, (B) two-dimensional force sensor DFM-20, (C) chamber, (D) peristaltic pump, (E) CENTR software, (F) optical microscope, (G) basic abrasive tool, (H) water inlet, (I) internal water injection abrasive tool, (J) femur head, (K) clamp, and (L) thermocouple.

The abrasion forces were measured using a two-dimensional force sensor DFM-20 equipped with UMT Bruker Tribometer. Penetration depth was measured using a step motor and CETR software. A force sensor in the Z-axis direction monitored a constant load. The entire measuring system is presented in Figure 3. The signal from the tribometer was recorded using the dedicated signal acquisition system. During the first stage of measurements, the cutting temperature was measured with a thermocouple. An acoustic emission sensor was used to assess the tissue structures’ potential changes during processing and the effects of transition between successive layers. Signals of the components of the total force, tangential force, movement range, cutting velocity, and tool position were measured in the following directions:

- Z-axis—normal force *F_N_* (N), tool position z (mm), penetration depth Δ*z* (mm), and penetration depth velocity *v_z_* (mm/min);
- Y-axis—tool position *y* (mm), movement range Δ*y* (mm), and velocity *v_y_* (mm/min);
- X-axis—tangential force *F_T_* (N), tool position *x* (mm), movement range Δ*x* (mm), and velocity *v_x_* (mm/min).

The analysis of the dynamics of the abrasion process was divided into several stages (see Table 3). In the first one, four samples of all types of granulation (from 46 to 150) were tested, for both materials (SiC and BFA), for pressure forces *F_N_* (from 5 to 20 N) in dry and wet conditions. Each test lasted 30 min with speeds *v_x_* = 240 mm/min, *v_y_* = 120 mm/min, and Δ*x,* Δ*y* = 1 mm. An external irrigation system was used in this test (see Figure 2B). This stage’s task was to detail the tools of the basic parameters, such as granulation and pressure force parameters. In the next step, an internal water irrigation system was used (see Figure 2C). The 10 tools of two types, BFA80 and SiC80, were prepared. Using the parameters *F_N_* = 10 N, *t* = 30 min, *v_x_* = 240 mm/min, *v_y_* = 120 mm/min, and Δ*x*, Δ*y* = 1 mm, the tangential forces *F_T_* during machining were then determined. This study finally showed which of the materials indicates better suitability for this machining type. The last measurement, which was used with the BFA80 tool with the parameters *F_N_* = 10 N, *t* = 10 min, *v_x_* = 240 mm/min, *v_y_* = 120 mm/min, and *v_y_* = 120 mm/min, was to show the nature of the changes in forces when reducing the machining range: Δ*x,* Δ*y* = 0.1–4 mm. The frequency of the movement in all steps was *f* = 1 Hz. The cutting mechanism used in this work reduces the problem to a linear, reciprocating motion. This mechanism can be compared to single-grain scratch tests [43]. Abrasion forces are related to ductile deformation [44], elastic deformation, chip forming, and friction [45]. The abrasion force results from the contact between the abrasion surface and the material. The surface contact leads to plastic deformation [44], chip formation, and the complicated phenomenon of friction [46]. A complex multi-grain abrasion process can be simplified using a single-grain perspective [44,47,48]. Considering microscopic cracking, crack formation, and chip formation, the entire cutting process can be divided into three phases: elastic deformation, pressure softening, and scratching [49]. When the grains and the workpiece surface come into contact during elastic deformation, the grain or workpiece deforms and generates frictional heat [50,51]. The process temperature was measured with a thermocouple to analyze the potential for thermal necrosis. In the next stage, an increase in chip thickness causes increased plastic deformation, inducing microcracks [52]. The relative motion of the grain produces tangential forces and shear stresses [53]. When a certain penetration depth is reached in the last stage, lateral cracks and chips of a certain depth are formed. Grain size and scratching velocity can have a significant effect on material removal and surface formation properties. To create an abrasive tool that allows for the processing of cartilage and bone tissue, appropriate guidelines for selecting cutting materials and the processing parameterization should be analyzed. Basic information on the machining efficiency dependence on the type of material processed was determined empirically following theoretical abrasion principles [54]. For most construction materials, machinability is defined as the physical relationship between process performance indicators and the mechanical properties of the materials being processed. The materials’ properties are determined by the intensity of stresses in the temperature and the speed range of abrasion deformation, geometry, and blunting of the tool grains. The abrasion process is determined by the analytical model of the estimated depth of cut [55]. The model considers the influence of the normal force acting on the cutting grain, the intensity of stresses in the shear zone of the processed material, the geometry of the cutting part of the abrasion grains, and the nature of the material’s behavior deformation zone. However, it should be noted that, in the case of materials such as cartilage, the abrasion process may differ diametrically from the previously described cases.

The friction coefficient was determined experimentally using the tribometer software. Multiple criteria of abrasion optimization presented by Kuo et al. [56] suggested that the soft material machining resulted in a higher coefficient of friction due to the higher plasticity. The tool wear measurements were conducted using a stereoscopic MOTIC SMZ-168-TL microscope, a TESCAN Vega 3 scanning electron microscope (SEM) with a secondary electron detector, an acoustic emission sensor, and the Kern ADB 200-4 weighting machine with high measuring accuracy (0.0001 g). Cartilage and bone tissue are easy to process, which results in the low wear of the abrasive tools. The tool life and the machining-process-related efficiency were most influenced by the accumulation of tissue on the grain surface, making further processing impossible. Therefore, the description of the results focuses on such an interpretation of the measurements. As mentioned in the introduction, one of the most common problems associated with cartilage and bone tissue processing is heat propagation due to friction. This phenomenon is evident during processes accompanied by the high speed of tool movement. In the study, the K type thermocouple (with an accuracy of ±2.2 °C) was placed inside the polyamide pinion next to the surface of the abrasive grain (see Figure 3). The temperature of the abrasive tool was analyzed in real time.

## 3. Results

### 3.1. Analysis of Cutting Forces

**Figure 4:**
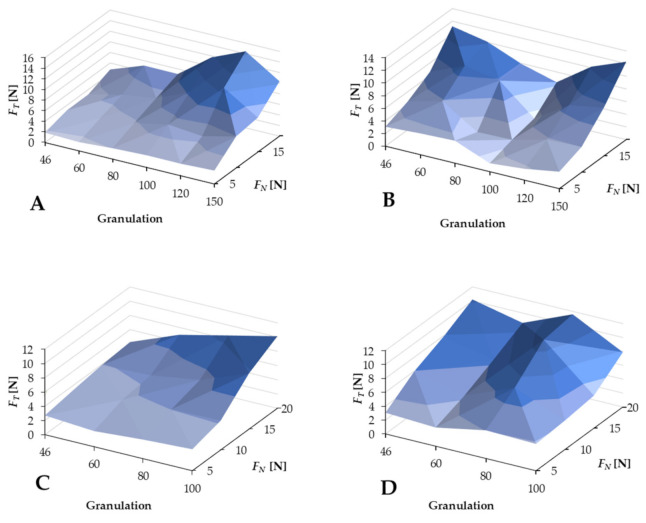
Results of the first stage of the machining tests: (**A**) BFA abrasive tools with external water injection, (**B**) SiC abrasive tools with external water injection, (**C**) BFA abrasive tools under dry conditions, and (**D**) SiC abrasive tools under dry conditions.

The results (see Figure 4) indicate that the tangential force *F_T_* increases as the regular force *F_N_* increases. This property applies to all tested cases, and the general values of *F_T_* forces are comparable, from *F_Tmin_* = 1.32 ± 0.01 N to *F_Tmax_* = 14.45 ± 0.01 N (see Table 4). A slight change was noticeable for the SiC100 abrasion tool operating in wet conditions. However, this may be related to the properties of the machined tissue. Significant differences were noticed in the analysis of the effect of granulation on the *F_T_* force value. For BFA-type abrasive tools (see Figure 4A,C), the reduction in the grain size causes an increase in the *F_T_* force value, which was noticeable for the normal force *F_N_* from 10 to 20 N. In the case of SiC abrasive tools (see Figure 4B,D), the visible effect of granulation on the increase in tangential force *F_T_* was not noticeable. Concluding from the above, with a constant value of the normal force *F_N_* (5, 10, 15, 20 N) set during the test, only the tools’ structure could influence the force *F_T_* values.

The presence of water allowed for lower values of *F_Tmin_* and *F_Tavg_* for both types of abrasion tools (see Table 4). The results also showed a less predictable cutting process with SiC grains. For BFA grains, there was a noticeable increase in *F_T_* with an increase in grain size and *F_N_* strength. In the case of SiC, the increase in grain size did not result in a marked increase in the force *F_T_* (there were variations for SiC60 and SiC100 in dry conditions and SiC80 and SiC 100 in wet conditions).

To analyze the results, it was necessary to calculate the total cutting force (according to Formula (1)) and the difference between the values of the entire cutting forces according to the following formula:

(1) $$\Delta F=F_{BFA_{wet}}-F_{BFA_{dry}}$$

**Figure 5:**
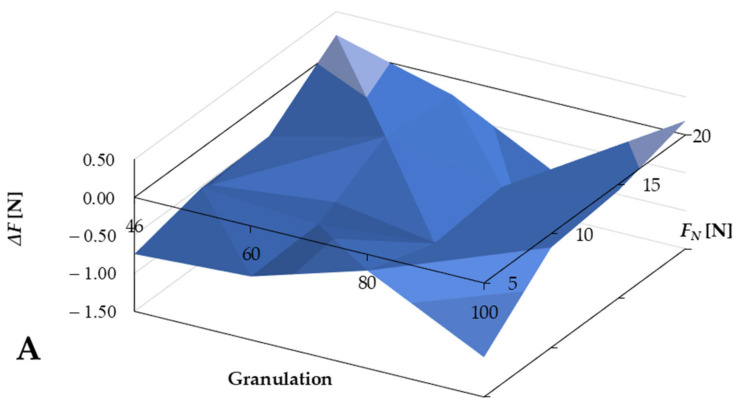

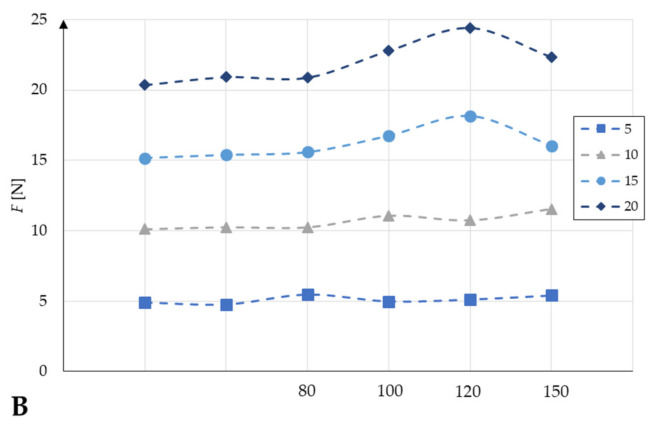
Difference between the values of the total cutting forces in wet and dry conditions for BFA abrasive tools: (**A**) force difference presentation and (**B**) total cutting forces for the BFA abrasive tool.

The results, shown in Figure 4A, indicate that the total cutting force *F* values are smaller for the wet conditions for forces below 15 N. In the range of 5–15 N *F_N_*, the grain size does not affect the results. The total cutting force for BFA in wet conditions is shown in Figure 5B. There was a noticeable effect of the normal force *F_N_*, which is several times greater than the tangential force *F_T_*, although the impact of granularity was also visible.

The value of the total force increases with the reduction in the grain size. However, attention should also be paid to the effect of grain size on the tangential force *F_T_* value, a component of the total force *F*, because it is the primary determinant of the machining process. Since the device must follow the surface structure and react to the phenomena occurring during machining, the actual pressure force *F_NR_* may differ slightly from the theoretical *F_N_*. This difference can reach as much as 0.3 ± 0.1 N, and for dry conditions, it can be about 0.05 ± 0.1 N. The return of force is also essential because, for BFA Wet and SiC Wet, these values are positive—consistent with the *F_N_* direction, which indicates the need to follow the machining. In BFA Dry and SiC Dry, these values were negative, indicating a lower intensity of the process. The average *F_T_* value for BFA was 0.75 ± 0.1 N and 1.5 ± 0.1 N for SiC. Therefore, the difference was significant and must have resulted from the geometry of the grain of the abrasion tool. The performance of the BFA tool was stable, and the standard deviation was only 0.2 N, while the SiC deviation was 0.8 N.

### 3.2. Analysis of Friction Coefficient

**Figure 6:**
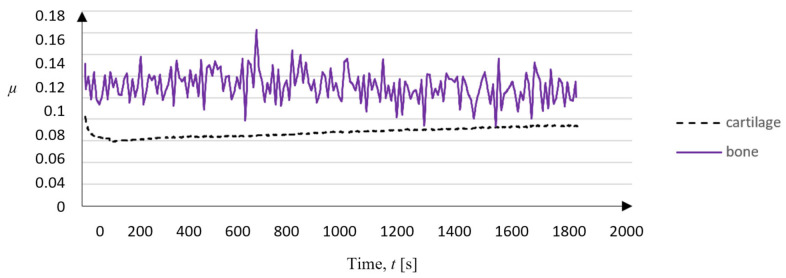
Coefficient of friction of periarticular tissues during erosion machining using the BFA80 abrasive tool with an internal water injection system.

Evaluation of the friction coefficient clearly shows that the average value of the coefficient depends only on the abrasive material (SiC or BFA) and working conditions (wet or dry). Additionally, the graph introduces the coefficient of friction obtained for machining bone tissue and cartilage tissue for the BFA80 tool during 10 processes using the internal water injection system (see Figure 2C). The mean value of µ for cartilage tissue under wet external conditions was *µ_c_ext_* = 0.45 ± 0.02 and that for cartilage tissue under wet internal conditions was *µ_c_int_* = 0.07 ± 0.02. The comparison between bone tissue *µ_b_int_* = 0.11 ± 0.02 and cartilage tissue *µ_c_int_* shows that both materials’ erosive machining process was similar, but the probability of tool wear increases when processing larger amounts of bone tissue. Both effects, obtained in water internal injection conditions, are shown in Figure 6.

### 3.3. Analysis of the Penetration Depth Process

The results indicate that the most significant depth was obtained with the use of abrasive tools with a grain size of 80 and a load *F_N_* of 5–10 N. In the case of the BFA tool, the obtained results were more readable, indicating a regularity of the process and higher durability. This was evidenced by the fact that, with the increase in force *F_N_* and granulation, the force *F_T_* increased, without fluctuations and disturbances (see Figure 4).

**Figure 7:**
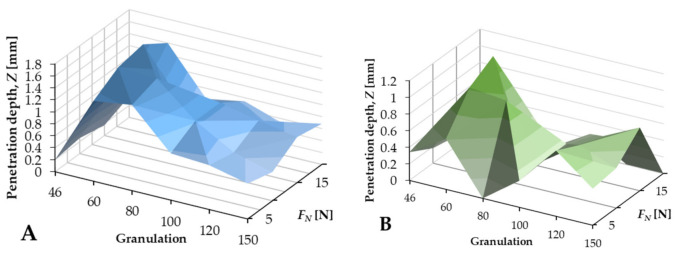
Penetration depth analyses of the BFA80 (**A**) and SiC80 (**B**) tools in wet conditions.

Table 5 shows the average values of the depth of cut for subsequent *F_N_* loadings. It is clear that, for the force *F_N_* = 10 N, the best results were obtained in the penetration. As strength increased, performance decreased. Comparing Figure 7 and Table 5, it should be concluded that the best results in the embedding process were obtained for the BFA tool with a granulation of 80 and a normal force at the level of 10 N.

**Figure 8:**
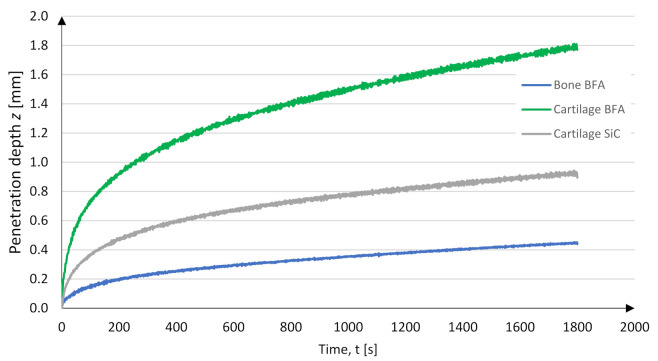
Penetration depth process of BFA80 and SiC80 in cartilage and bone tissue.

The above data represents the measurements performed during the processing of the total periarticular tissue. From the obtained data, it was possible to distinguish information on the process of embedding in the cartilage and bone tissue. Figure 8 shows the course of the cutting process in bone and cartilage tissue. The difference between the penetration efficiency for the type of BFA and SiC tool and the type of material was noticeable. The mean depth value for BFA80 during *t* = 30 min was *z_avg_b_* = 0.49 mm for bones and *z_avg_c_* = 0.83 mm for cartilage tissue (see Figure 8).

### 3.4. Evaluation of Tool Wear and Machining Efficiency

**Figure 9:**
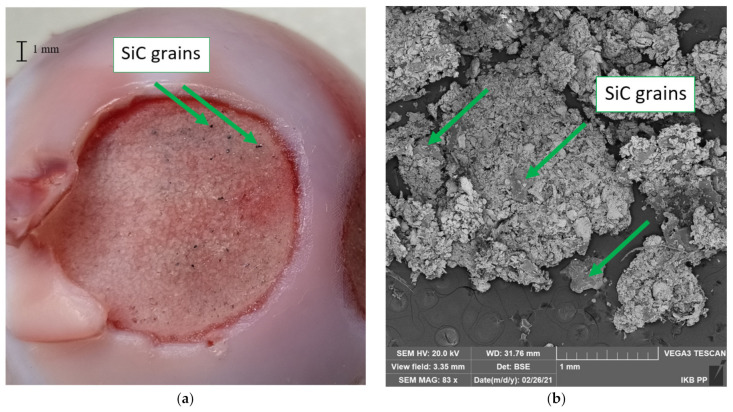
After machining, the femoral head (**a**) and a SEM photo (**b**) of chips taken from the SiC80 tool with marked SiC grains.

Twenty measurements were carried out in the tool wear analysis. They included the cutting of cartilage and bone tissue of the femoral head. The test parameters were, for the BFA80 (5 wet and 5 dry measurements) and SiC80 (5 wet and 5 dry measurements) tools, Δ*x,* Δ*y* = 2 mm, *v_x_* = 4 mm/s, *v_y_* = 2 mm/s, *F_N_* = 10 N, and *t* = 5000 s. A water internal injection system was used. A wider range of Δ*x* and Δ*y* movement allowed for faster machining. In the case of the SiC80 tools, clear grain residues were visible on the surface of the bone tissue and among the chips (see Figure 9). In the case of the BFA tool, no grain residue was noticed.

The tools after machining indicated a more significant loss of grains in the case of dry testing samples. Some broken grains were deposited on the chips, and some were stuck in the tissue (see Figure 9). During wet tests, few grains were noticeable during the analysis of the sediment in the sample washing fluid. A more significant number of crushed grains was noted in the operation of the SiC abrasion tool. Another critical issue is to discuss the process of recessing the abrasion tool in the tissue by analyzing the value of cavity z at time t and the increment of cavity Δ*z_t_* with time. The graphs show the machining of the femoral head using the BFA80 abrasion tool, with machining parameters Δ*x*, Δ*y* = 1 mm, *v_x_* = 4 mm/s, *v_y_* = 2 mm/s, and *F_N_* = 10 N. 

**Figure 10:**
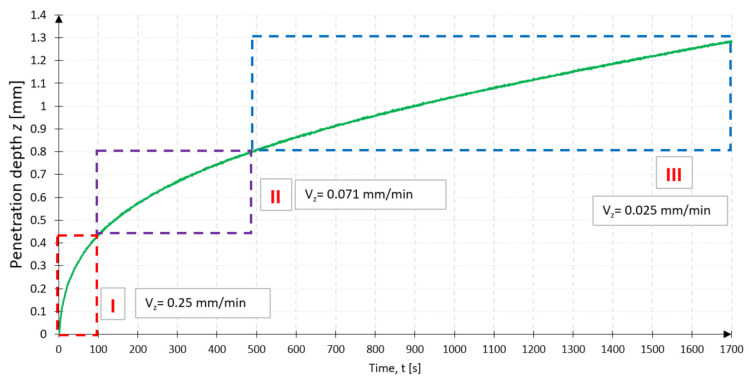
The penetration depth and speed of insertion of the tool during the three stages of machining.

**Figure 11:**
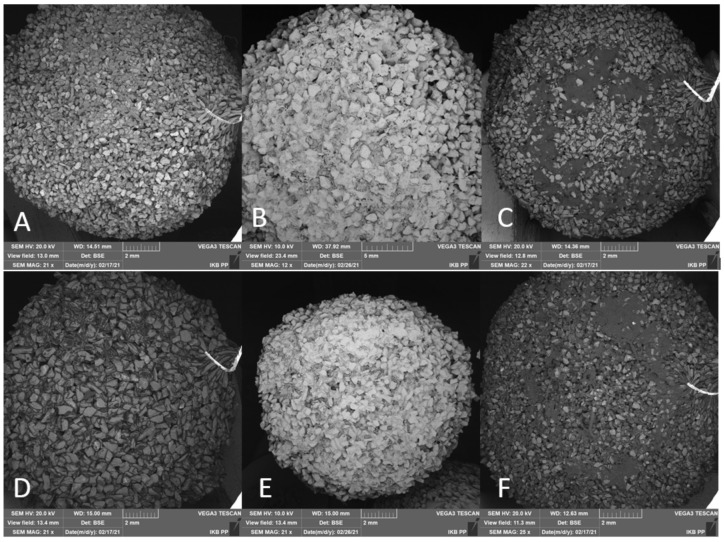
Tool front view: (**A**) BFA E80 before machining, (**B**) BFA E80 after machining with water, (**C**) BFA E80 after machining without water, (**D**) SiC E80 before machining, (**E**) SiC E80 after machining with water, and (**F**) SiC E80 after machining without water.

While analyzing the data, three stages of the tools work were distinguished. Stage I (red rectangle, Figure 10) consisted of working at time *t* = 100 s, where the increase in penetration was Δ*z* = 0.435 mm. This process was also supported by water supplied within the working space, thoroughly washing the material cut in the initial phase. At this time, there was also the most dynamic decrease in penetration value over time Δ*z_t_* (see Figure 10), which can be explained by the intensive removal of material, some of which accumulates on the surface of the tool, reducing its working surface. At this stage, the insertion speed was *v_z_* = 0.25 mm/min. Stage II (purple rectangle, Figure 9) consisted of work from *t* = 100 s to *t* = 500 s, where the depth increase was Δ*z* = 0.372 mm. In this process, a gradual, non-linear stabilization was noticeable, which reduces the entire system to the penetration speed at the level of *v_z_* = 0.071 mm/min, which is already a significant decrease concerning the value of Stage I. An increase in the depth with time Δ*z_t_* significantly changed the dynamics to stabilize. Stage III (blue rectangle, see Figure 9) covered the time from *t* = 500 s to the end of the process, during which the abrasion tool was entered with the value of Δ*z* = 0.478 mm. Over time, the depth increment over time Δ*z_t_* stabilized and caused the abrasion tool to penetrate linearly at a speed of *v_z_* = 0.025 mm/min. The tool gradually sinks into the surface of the tissue, but the amount of material that obstructs the tool movement and the reduced working space results in a very high-performance limitation. SEM photographs of the BFA and SiC tool faces were taken before machining, after dry machining, and after machining in an aqueous environment (see Figure 11). Samples of two types of material and with a different grain size were tested. The assessment included an analysis of SEM photographs and the measurement of the tool’s weight before and after machining. Visual assessment of the chips showed possible abrasive grains in the cooling fluid.

Before machining, both the BFA and SiC surfaces (see Figure 11A,D) are characterized by a homogeneous structure without any damage or losses in the grain system. After performing cutting treatment on a pig femoral head sample during *t* = 60 min, with a pressure *F_N_* =10 N, a feed Δ*x*, Δ*y* = 1 mm, and speeds *v_y_* = 4 mm/s and *v_x_* = 2 mm/s, the expected results were obtained. Instruments operating in the water internal injection mode (see Figure 11B,E) show traces of cartilage and bone tissue in the grooves between the grains in a small amount. While working in a dry environment (see Figure 11C,F), tissue visibly stuck to the abrasive tool surface, creating a barrier that prevented further tool operation. In the wet process, the abrasion tool penetrated much better, and in the case of dry operation, the structure was glued faster.

### 3.5. Evaluation of Chip Forming Mechanism

**Figure 12:**
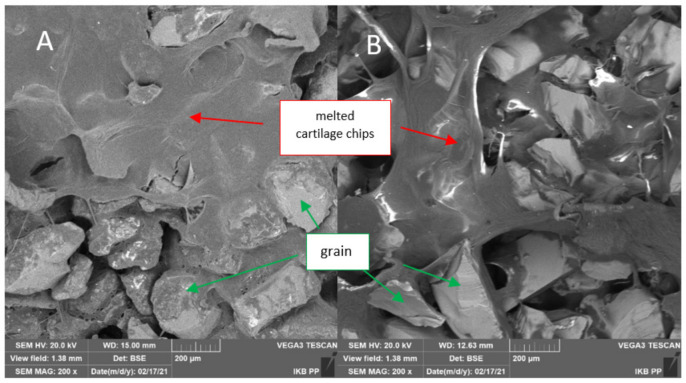
The effects of machining of cartilage with a tool in dry conditions: (**A**) BFA and (**B**) SiC.

**Figure 13:**
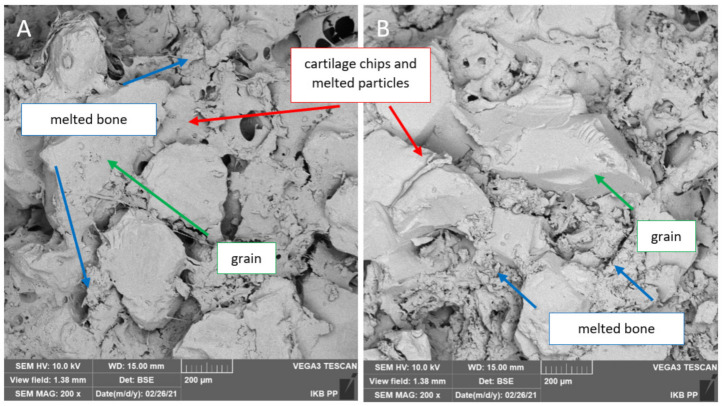
The effects of cartilage and bone machining with a tool in dry conditions: (**A**) BFA and (**B**) SiC.

The chip formation analysis was based on the test results of 96 samples used during the entire study process. To evaluate the chips, optical analysis with a microscope and SEM imaging were used. In dry conditions, the abrasive tool was stuck with cartilage tissue without a clear separation of the material for all grain sizes and both materials. The chips did not take any characteristic shapes, creating a slime sticking to the grains (see Figure 12). This phenomenon reduced machining efficiency, preventing the tool from reaching the bone tissue space. The tool’s weight increased significantly, and the residual material was visible (see Figure 11C,F). In the simultaneous processing of cartilage and bone tissue, the residual cartilage material limited the processing of bone tissue, although it was slowly mixed with bone fragments, filling the spaces between the grains (see Figure 13). 

**Figure 14:**
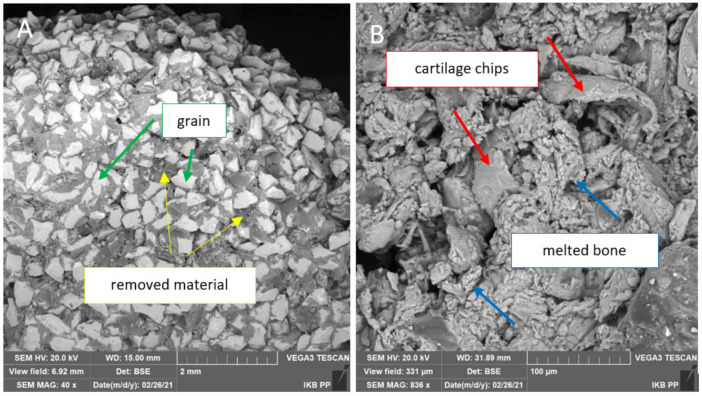
The effects of machining of cartilage and bone within wet conditions: (**A**) the tool surface and (**B**) the chips after wet machining.

Different results were obtained with wet machining. While cutting the cartilage tissue, there was a clear separation of tissue fragments from the surface, and the pieces in the form of chips with a continuous, smooth shape and undergoing constant transformation flowed with the water into the reservoir. These liquid chips took the shape of threads and ribbons of various lengths. Figure 14 shows the chips resulting from the cartilage treatment after drying at 30 °C. Most of the shavings were ground. A few remained unchanged (shown in Figure 14B). The tool did not stick to the wet machining, and only a few contaminants were visible (see Figure 14A).

In the chips produced during the machining of bone tissue, elementary, detached, single, unbound chips were obtained, with a rough surface, characterized by low plasticity (see Figure 14B). It should be noted that both bone chips were ground continuously because the cutting surface was in constant contact with the processed material. The chip forming stage can be divided into the single-pass formation of grain, melting due to multiple tool movements, and the final chips exited from the workspace. Regardless of the grain size, this process resulted in chips of sizes from 5 to 50 µm. Temperature measurements during the machining process under all conditions and procedures showed no increase in heat propagation. In dry conditions, the temperature remained constant with fluctuations of ±0.6 °C. The temperature initially dropped from 21 °C to stay regular with less ±0.3 °C fluctuations in wet conditions.

## 4. Discussion and Conclusions

In this work, an abrasion machining process of cartilage and bone tissue was proposed and experimentally tested. The cutting device used the abrasion grains’ undefined geometry to cut in any direction and on an undefined surface. The machining was carried out following the pressure force’s specified parameters, cutting speed and grain granulation, in the range of two materials in dry and wet conditions. A set of tools was prepared based on two abrasives, brown fused alumina and silicon carbide, with five granularities to validate the assumptions.

The developed abrasion machining process with the BFA tool dedicated to machining periarticular tissue assured advantageous effects in tool life for cutting conditions. The proposed method of joint machining concerned mainly the presented tool shape, but as a result of appropriate modification of the input data, it can be easily adapted to the machining of any form. The proposed machining method is comparable to the previously used methods and research results on cutting, milling, and drilling. Most importantly, the treatment did not increase the processed tissue’s temperature in the proposed basic version, and there was no thermal necrosis.

To create an abrasive tool that allows for cartilage and bone tissue processing, appropriate guidelines for selecting cutting materials and parameterization should be analyzed. Basic information on the machining efficiency dependence on the type of material processed can be determined empirically following theoretical abrasion principles [45]. However, both cartilage and bone tissue are characterized by variable mechanical properties due to the heterogeneity of the cell structure, changes in density, and local (individual) conditions caused by injuries and diseases. For most construction materials, machinability is defined as the physical relationship between process performance indicators and the materials’ mechanical properties being processed. 

One of the parameters is the cutting resistance expressed in N/mm^2^ or MPa. This parameter is defined as the ratio of the main component cutting forces to the cross-sectional area of the cutting layer. In the analysis, the tool diameter D1 was assumed as the maximum cutting width. Half of the mean grain size was assumed for the depth of cut for the extreme grain sizes. The presented assumptions allow one to determine the most significant value of cutting resistance (see Table 6). The obtained results indicate a wide discrepancy between the minimum and maximum values. The differences between the machining methods correspond to the cutting forces, which is discussed in Section 3.1. Compared to other materials, periarticular tissues are characterized by low cutting resistance values. For example, for similar cutting parameters for steel, we obtain a cutting resistance value over 1000 MPa [57], 50–400 MPa for wood [58], and 100–300 MPa for HDPE polymers [59]. As can be seen, the obtained results are much lower, which proves the high workability of periarticular tissues. Their mechanical and structural properties (the high porosity of the bone tissue and the elasticity of the cartilage tissue) may contribute to this. These results, however, need to be supplemented with comparative measurements using the research methodology based on orthogonal cutting.

**Figure 15:**
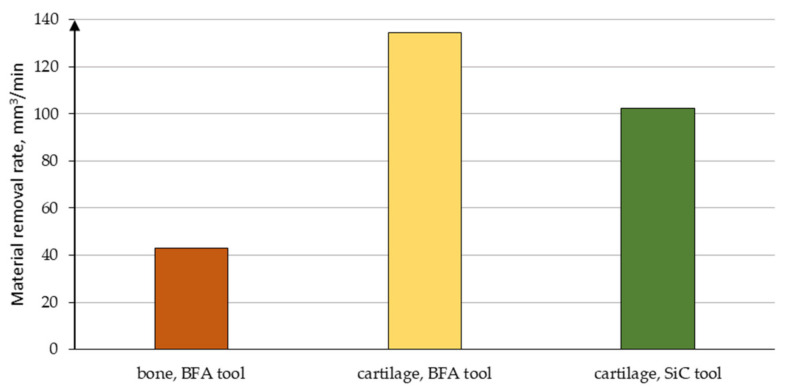
Material removal rate obtained while conducting studies.

The materials’ properties are determined by the intensity of stresses in the temperature and the speed range of the abrasion deformations, geometry, and blunting of the tool grains. The abrasion process is determined by the analytical model of the estimated depth of cut [45]. The model considers the influence of the normal force acting on the cutting grain, the intensity of the stresses in the shear zone of the processed material, the geometry of the cutting part of the abrasion grains, and the nature of the material’s behavior deformation zone. However, it should be noted that, in the case of materials such as cartilage, the abrasion process may differ diametrically from the previously described cases. The obtained experimental results show that the theoretical penetration into the cartilage tissue is carried out much faster than that into the bone tissue due to the low hardness of this material (see Figure 15). The results presented below clearly indicate a potentially more practical application of the BFA-type abrasive tool due to its greater material removal ability. Moreover, when water injection is used, the material removal rate increases.

Evaluation of the friction coefficient clearly shows that the average value of the coefficient depends only on the abrasive material (SiC or BFA) and the working conditions (wet or dry) for the contact of the abrasive tool with cartilage tissue, which are similar for all types of processing (see Figure 14). Comparing the test results (obtained for external water injection and dry conditions) with the coefficient of friction analyses for cartilage tissue contact with Al_2_O_3_, CoCr, SS, and UHMWPE, similar results are found [60]. Additionally, the graph introduces the coefficient of friction obtained for machining bone tissue and cartilage tissue for the BFA80 tool in 10 processes using the internal water injection system.

**Figure 16:**
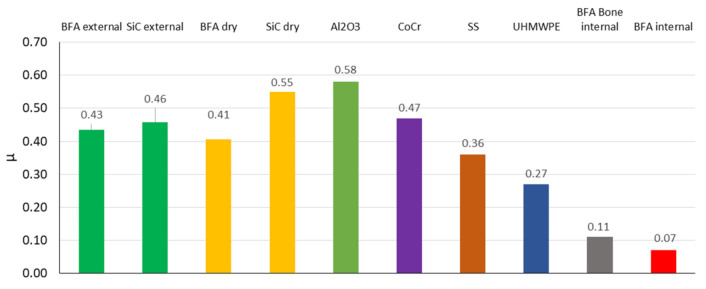
Comparison of coefficient of friction between abrasion material and periarticular tissues for current test results and other materials [61].

The summary of studies indicates the reproducibility of the results concerning three other studies. In the studies by Hayden et al. [61] and Chan et al. [60], the bone friction coefficient *µ_c_int_* increases with the test duration. The bone friction coefficient *µ_b_int_* stabilizes in line with the results obtained by Krishnan et al. [62], although it is marked by a significant fluctuation in value, which characterizes the cutting process. Both effects, obtained in water internal injection conditions, are shown in Figure 16.

**Figure 17:**
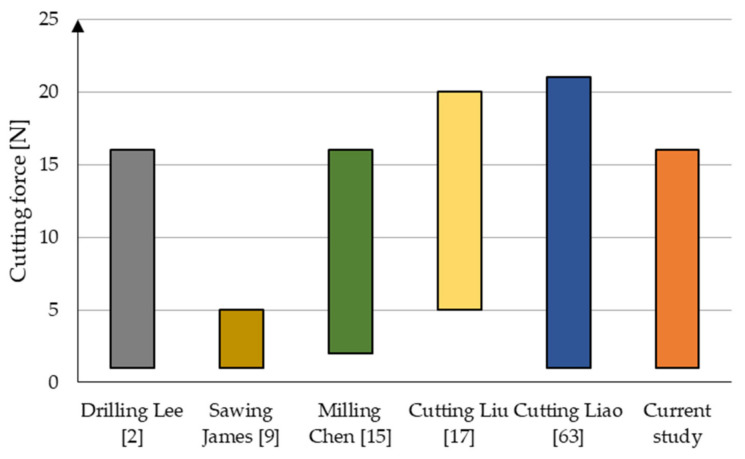
Comparison of cutting force for the tested tool and data from the literature [2,9,15,17,63].

The graph (see Figure 17) provides comparative information with other machining methods reported in the literature. Cutting force is the resistance of the material against the intrusion of the cutting tool. It is the basic parameter characterizing the arrangement of the material-cutting tool, so it was selected as a parameter comparison. There are several tests against which cutting force values can be compared. In the drilling analysis by Lee et al. [2], the *F_c_* value coincides with the test results, despite the differences in the method used. A similar result was obtained in studies by Chen et al. [15]. This result can be disputed because of the tool’s similar construction and operating principles, which are the drill and the cutter, in bone tissue processing. In the other three studies, the results slightly differ. The cutting force value is within the indicated test range, proving that the presented solution works correctly. There are no specific considerations in the case of studies on the abrasive processing of bone and cartilage tissue. However, significant comparative conclusions can be drawn from the available studies. In all studies, particular attention was paid to the heat generated during tool operation, caused by the tool’s high speed of feed and rotation. Increasing the amount of coolant positively influenced the results, minimizing the possibility of necrosis. In the above tests, no temperature increase was noted due to the use of a coolant. In a study by Mizutani et al. [18], heat emission rose clearly after about 60 s of tool operation. The difference in both tests was noticeable, resulting from the tool’s low speed and direct injection of water into the machining space, based on Zhang et al. [19]. Thus, the reduction in the frequency of tool movement and the introduction of constant cooling, as the study shows, are reflected in external studies.

The analysis of the chip shape and of the mechanism of their formation yields similar results to research carried out by Bai et al. [64]. The authors indicate that the chip formation process is highly dependent on the anisotropy structure of the bone tissue. The material is characterized by high brittleness and susceptibility to cracking. These observations confirm the results of the above studies presented in Figure 13A,B and Figure 14B. The bone tissue was broken into smaller fragments during subsequent passes of the tool, but with similar characteristics.

A limited number of substantive studies have been carried out, allowing for the precise characterization of the treatment of articular surfaces using specific methods. The machining process method presented in this study may become a starting point for more extensive research related to cartilage tissue machining and its accompanying processes. It should be noted that this type of research has not been carried out before. Therefore, the study covered a wide range of constant and variable parameters, which necessitated a significant number of measurements. The results highlight the potential of the proposed tool design concept in designing new methods for machining periarticular tissues. Further plans include the development of appropriate machining kinematics and the development of a universal tool geometry.

## Figures and Tables

**Table 1:** Characteristics of grains used for abrasive tool production.

Material	Grain Number	Grain Size (μm)		
		Min	Max	Mean Grain Size *b* (µm)
Brown fused alumina and silicon carbide	F46	355	425	390
	F60	250	300	275
	F80	180	212	196
	F100	125	150	137.5
	F120	106	125	115.5
	F150	75	106	90.5

**Table 2:** Abrasive tool dimensions.

Symbol	*D*1	*D*2	*L*1	*L*2	*b*
Description	Abrasive Tool Diameter	Pinion Diameter	Pinion Length (External Water)	Pinion Length (Internal Water)	Mean Grain Size
Value	16 mm	12 mm	30 mm	50 mm	Depends on granulation type

**Table 3:** Measurement of abrasion process dynamic parameters.

Cutting Speed, mm/min		Movement, mm		Time, min	Load, N	Granulation	Grain Material	Environment	Water Injection Type	Number of Samples
*v_x_*	*v_y_*	Δx	Δy	*t*	*F_N_*					
Basic Details Stage										
4	2	1	1	30	5, 10, 15, 20	46, 60, 80, 100, 120, 150	BFA, SiC	Wet and dry	External	384
Material Determination Stage										
4	2	1	1	30	10	80	BFA, SiC	Wet	Internal	20
Machining Range Stage										
4	2	0.1, 0.25, 0.5, 1, 2, 3, 4, 5	0.1, 0.25, 0.5, 1, 2, 3, 4, 5	10	10	80	BFA, SiC	Wet	Internal	16

**Table 4:** Maximal, minimal, and average tangential force results for different abrasive tool materials and conditions.

Tangential Force Type	BFA Wet	SiC Wet	BFA Dry	SiC Dry
*F_Tmax_*, N	14.45	11.20	10.12	11.58
*F_Tmin_*, N	1.65	1.32	2.19	2.68
*F_Tavg_*, N	5.51	5.68	4.80	6.46

**Table 5:** The average and maximal penetration depth of the BFA80 and SiC80 tools.

Tool	Load *F_N_* (N)			
	5	10	15	20
	Average Penetration Depth, *z_avg_*			
BFA80 wet	0.85	0.93	0.85	0.72
SiC80 wet	0.50	0.61	0.59	0.34
	Maximum Penetration Depth, *z_max_*			
BFA80 wet	1.43	1.6	1.68	1.4
SiC80 wet	0.79	1.02	1.19	0.42

**Table 6:** The minimal and maximal cutting resistance for wet and dry machining conditions.

Tangential Force Type	BFA Wet	SiC Wet	BFA Dry	SiC Dry
Cutting Resistance Minimum, *kc_min_* (MPa)				
*F_Tmax_*	4.63	3.59	3.24	3.71
*F_Tmin_*	0.53	0.42	0.70	0.86
*F_Tavg_*	1.77	1.82	1.54	2.07
Cutting Resistance Maximum, *kc_max_* (MPa)				
*F_Tmax_*	19.01	14.74	13.32	15.24
*F_Tmin_*	2.17	1.74	2.88	3.53
*F_Tavg_*	7.25	7.47	6.32	8.50

## Data Availability

The data presented in this study are available on request from the corresponding author. The data are not publicly available due to the large amount of data.
